# Lenalidomide and Rituximab Regimen Combined With Intravitreal Methotrexate Followed by Lenalidomide Maintenance for Primary Vitreoretinal Lymphoma: A Prospective Phase II Study

**DOI:** 10.3389/fonc.2021.701507

**Published:** 2021-06-24

**Authors:** Yan Zhang, Xiao Zhang, Dongmei Zou, Jingjing Yin, Li Zhang, Xuan Wang, Congwei Jia, Wei Wang, Danqing Zhao, Daobin Zhou, Wei Zhang, Meifen Zhang

**Affiliations:** ^1^ Department of Hematology, Peking Union Medical College Hospital, Chinese Academy of Medical Sciences & Peking Union Medical College, Beijing, China; ^2^ Department of Ophthalmology, Peking Union Medical College Hospital, Chinese Academy of Medical Sciences & Peking Union Medical College, Beijing, China; ^3^ Department of Hematology, Xuanwu Hospital, Beijing, China; ^4^ Department of Hematology, Beijing Hospital, Beijing, China; ^5^ Department of Clinical Laboratory, Peking Union Medical College Hospital, Chinese Academy of Medical Sciences & Peking Union Medical College, Beijing, China; ^6^ Department of Pathology, Peking Union Medical College Hospital, Chinese Academy of Medical Sciences & Peking Union Medical College, Beijing, China

**Keywords:** primary vitreoretinal lymphoma (PVRL), rituximab, lenalidomide, intravitreal methotrexate injection, safety

## Abstract

Primary vitreoretinal lymphoma (PVRL) is a rare variant of primary central nervous system (CNS) lymphoma, for which currently there are no optimal treatment options. This prospective single-center study enrolled immunocompetent patients with newly diagnosed PVRL between August 2018 and January 2020. Patients received local and systemic therapies: intravitreal methotrexate (MTX, 400 μg, 0.1 mL) injections for 1 year (total 16 injections) and six cycles of the rituximab (375 mg/m^2^ on day 1) and lenalidomide (25 mg on day 1–21; R2) regimen. Lenalidomide was maintained for 2 years in patients who had achieved a response. We enrolled 11 patients with a mean age of 58 (range, 48–70) years, of which 10 achieved complete remission at the first evaluation. The median follow-up period was 18.3 (range, 10.6–27.8) months, and the median progression-free survival was 12.7 months. Moreover, a total of eight patients relapsed. The most common adverse event (AE) was neutropenia, which occurred in seven patients (63.6%), followed by grade 3 ocular toxicities, including cataract formation, in six patients (54%). These findings suggest that the R2 regimen combined with intravitreal MTX, followed by lenalidomide maintenance, is a safe option for PVRL with moderate efficacy. This trial is registered with ClinicalTrials.gov (number NCT 03746223).

## Introduction

Primary vitreoretinal lymphoma (PVRL) or primary intraocular lymphoma is a rare variant of primary central nervous system lymphoma (PCNSL). The lesions are usually located in the vitreous, retina, choroid, and the iris or the optic nerve or both. The typical histologic subtype is diffuse large B-cell lymphoma. PVRL has a high association with the CNS. Approximately 20% to 25% of patients with PCNSL exhibit ocular involvement at the time of initial diagnosis, and 50% to 80% of patients eventually develop CNS involvement during the follow-up period (1, 2).

The best therapeutic strategy for PVRL without CNS involvement remains undefined because of the paucity of large-scale and high-quality trials. Currently, there are two distinct treatment approaches. The first consists of intensive chemotherapy, such as that used for CNSL including high-dose methotrexate (MTX)-based chemotherapy with or without whole-brain radiotherapy, which is used for PCNSL (3, 4). The second approach consists of local ocular treatments, including ocular radiotherapy, intravitreal chemotherapy with MTX, or both (5, 6). Recent studies have demonstrated that CNS relapse might not be prevented by local treatment alone, and systemic intensive regimens are related to severe hematological toxicities (7).

Lenalidomide is an immunomodulator that has demonstrated good response rate in relapsed/refractory PCNSLs and is well tolerated (8, 9). The combination of rituximab and lenalidomide (R2 regimen) has been widely used in multiple lymphomas (10, 11). To balance the adverse events (AEs) of chemotherapy and achieve sustained control of lymphoma, we conducted a single-arm, phase II trial for newly diagnosed B-cell PVRL to evaluate the efficacy and safety of the R2 regimen with intravitreal MTX.

## Patients And Methods

### Study Design and Participants

This prospective single-center, open-label, phase II trial assessed the efficacy and safety of intravitreal MTX combined with the R2 regimen followed by lenalidomide maintenance in patients with PVRL. Eligible patients received MTX intravitreally combined with the R2 regimen as induction therapy, followed by lenalidomide monotherapy for maintenance.

All patients signed a formal consent form before enrollment. The study conformed to the Declaration of Helsinki and was approved by the Institutional Review Board of Peking Union Medical College Hospital. This trial is registered with ClinicalTrials.gov (NCT04257656).

#### Patients

Immunocompetent patients aged 18–75 years with newly diagnosed B-cell PVRL were eligible for inclusion. In this trial, B-cell PVRL was diagnosed using the following parameters: 1) clinical manifestations: typical vitreous opacities, subretinal lesions, or both; 2) detection of aqueous humor or vitreous cytokines: interleukin (IL)-10/IL-6 > 1; 3) pathological examination: vitreous cytology showing malignant lymphoma cells; 4) vitreous cell gene rearrangement: IgH, Igκ, or Igλ (+); and 5) vitreous flow cytometry: B-cell lymphoma biomarkers in patients without evidence of CNS lymphoma before the onset of the eye lesion. Patients who fulfilled items 1 and 2 and two of items 3, 4, or 5 were considered positive for the condition and were diagnosed with PVRL.

The other inclusion criteria were as follows: Eastern Cooperative Oncology Group performance status ≤ 2, creatinine clearance rate ≥ 60mL/(min·1.73m^2^), bilirubin < 2 upper normal limit (UNL), and alanine transaminase < 3 UNL. The exclusion criteria were PCNSL with concurrent eye involvement, systemic lymphoma with eye involvement, active hepatitis virus B, and hepatitis virus C infection.

At the time of diagnosis, the following baseline staging procedures were performed: physical examination; biochemical serum profile; human immunodeficiency virus, hepatitis B virus, and hepatitis C virus serological assessments; gadolinium-enhanced whole-brain magnetic resonance imaging (MRI); whole body ^18^FDG-positron emission tomography or contrast-enhanced thorax-abdomen-pelvis computed tomography; ophthalmological assessment (including slit-lamp examination, fundoscopy, B-scan ultrasound, optical coherence tomography); cerebrospinal fluid (CSF) examination (cytological examination, biochemical examinations, and flow cytometry).

### Treatment and Evaluation

MTX (400 μg, 0.1 mL) was intravitreally administered four times weekly as a local therapy and then two times biweekly, followed by 10 times monthly. The entire duration of the intravitreal injections was 12 months, and a total of 16 injections were administered.

The R2 regimen was administered as a systemic therapy: rituximab 375 mg/m^2^ intravenously on day 0 per cycle along with lenalidomide 25 mg daily orally for 21 days per cycle, and each cycle lasted 28 days. An interim evaluation was conducted after three cycles, and patients showing a response or stable disease received three more courses of the same treatment regimen. A total of six cycles were administered as induction therapy. Patients with complete response (CR) and partial response (PR) without persistent iatrogenic side effects were eligible for maintenance therapy. This therapy involved 24 cycles of lenalidomide 25 mg daily for 21 days every 4 weeks.

Repeat imaging, lumbar puncture, and ophthalmological assessment were performed at the interim of induction therapy; completion of chemotherapy; every 3 months during lenalidomide maintenance for 2 years, every 6 months for 3 years, and annually for 5 years. Radiographic responses were graded as CR, PR, and progressive disease according to the International PCNSL Collaborative Group (12). Intraocular CR was defined as the disappearance of the intraocular lesion and undetectable IL-10 concentration in the aqueous humor. Intraocular relapse was defined as the appearance of a new lymphoma lesion of the retina or vitreous with a detectable IL-10 concentration in the aqueous humor (≥5 pg/mL).

### Outcome

The primary endpoint was 2-year progression-free survival (PFS), and the secondary endpoints were toxicity, overall survival (OS), relapse rates, and neurotoxicity. The final follow-up date was October 30, 2020.

### Statistical Analysis

Statistical analysis was performed to determine whether this regimen can improve the PFS. Using a one-sample chi-square test with a one-sided significance level of 0.20, 47 evaluable patients would have 87% power to detect a 20% relative improvement in the 2-year PFS compared with a historical control (60% *vs.* 80%). AEs were graded using the Common Terminology Criteria for Adverse Events v. 4.0 (CTCAE4.0) of the National Cancer Institute Common Toxicity Criteria.

The Kaplan-Meier method was used to estimate the PFS and OS from the date of diagnosis. PFS was defined as the time from receiving treatment to disease progression or death from any cause, whichever came first. Patients who were alive at the last follow-up were analyzed as censored observations for OS, and those who were alive at the last follow-up without disease progression were analyzed as censored observations for PFS. Statistical analysis was performed using the Statistical Package for the Social Sciences 25.0 software (SPSS Inc., Chicago, IL, USA).

## Results

**Figure 1:**
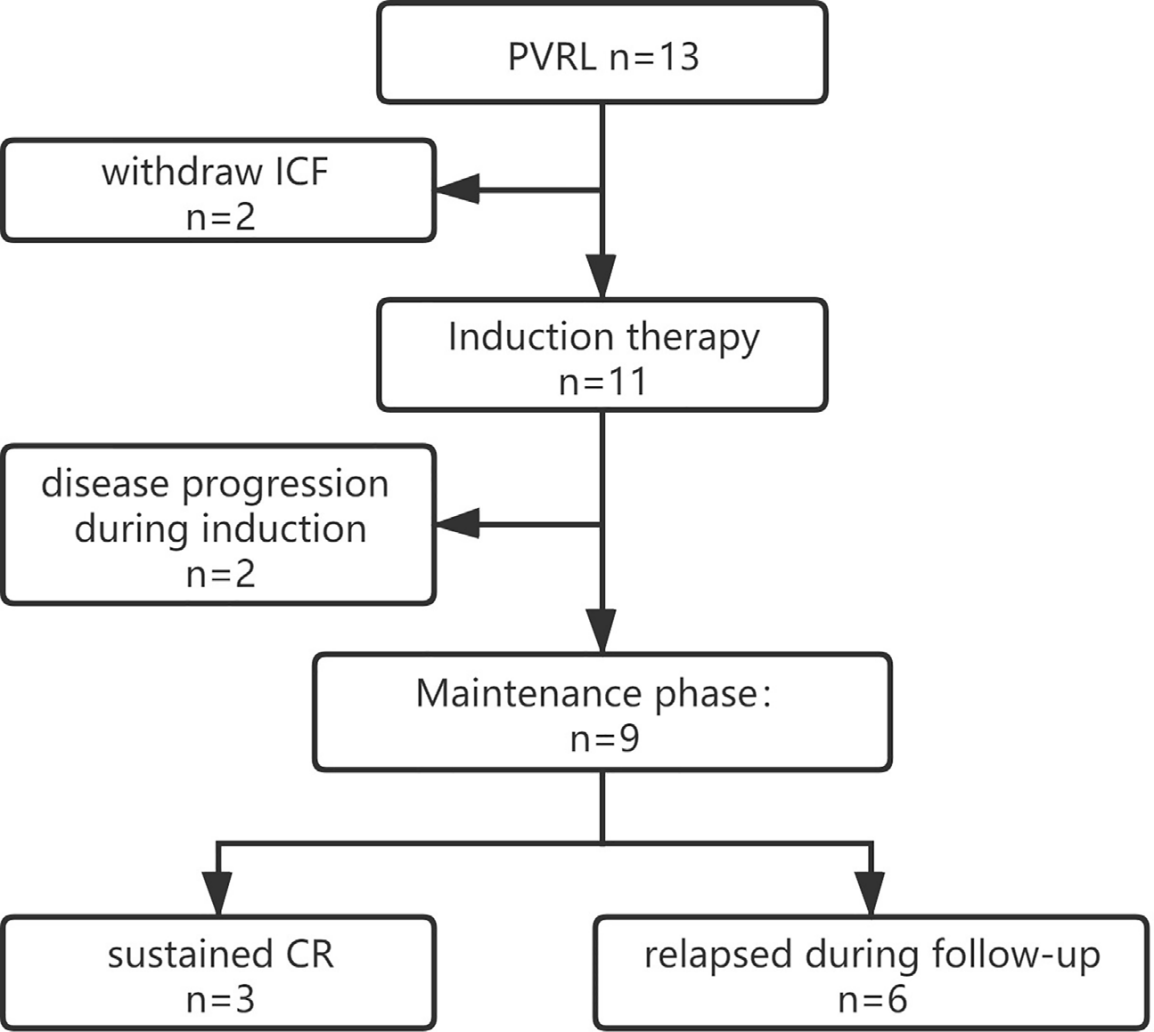
Trial profile. ICF, Informed Consent Form; CR, complete remission; PVRL, primary vitreoretinal lymphoma.

We enrolled 13 patients from September 2018 to January 2020 for PFS analysis (**Figure 1**), and 11 patients were administered the treatment per protocol and were assessed. Of these, eight and three patients were female and male, respectively, with a median age of 58 (range 48–70) years. Decreased visual acuity, blurred vision, and floaters were the most common symptoms in patients (45.5%, 36.4%, and 36.4%, respectively). The median interval from onset to diagnosis was 12 months (interquartile range [IQR], 3–12 months). The clinical characters were showed in **Tables 1** and **2**.

**Table 1:** Clinical characteristics of 11 PVRL.

Clinical characters	n,%
Age(median)	58 (48–70)
>60(n,%)	5, 45.5%
Bilateral Eyes involvement	7, 63.6%
Vitreous or retina samples	
Positive Cytology	8, 72.7%
Positive FCM	7,63.6%
Positive Ig rearrangement	6/9,66.7%
CSF examination	
IL-10 elevation(>5pg/ml)	11,100%
CSF MYD88 mutation	3/5,66.7%
Treatment before diagnosis	
Local steroids	5, 45.5%
Systemic steroids	3, 27.3%
Local and Systemic steroids	1, 9.1%
No treatment	2, 18.2%

CSF, cerebrospinal fluid; FCM, flow cytometry; Ig, immune globulin; IL-10, interleukin-10; MYD, myeloid differential protein.

**Table 2:** Patient characteristics, clinical findings, response and disease free survival.

Case	Age/sex	Primary site	interim evaluation after 3 cycles	End evaluation after 6 cycles	Relapse site	PFS(months)	OS(months)
1	F/58	Bilateral	CR	CR	Left eye	13.0	26.6
2	F/48	Right eye	CR	CR	Left frontal lobe	10.0	27.6
3	F/50	Left eye	CR	CR	Bilateral frontal lobes,basal ganglia	25.4	27.8
4	F/61	Left eye	CR	CR	Right eye	20.9	25.3
5	F/61	Bilateral	CR	CR	Left eye	12.7	22.0
6	M/58	Bilateral	PD	NA	Right precentral gyrus	1.2	17.6
7	M/48	Left eye	CR	CR	Bilateral precentral gyrus	9.2	18.3
8	M/52	Bilateral	CR	CR	NA	16.0	17.5
9	F/65	Bilateral	CR	CR	NA	12.7	15.3
10	F/48	Bilateral	CR	CR	NA	11.0	11.2
11	F/70	Bilateral	CR	CR	Bilateral corpus callosum, cerebellum,lateral ventricle	8.2	10.6

M, male; F, female; CR, complete remission; PD, progressed disease; NA, not Applicable.

### Response and Relapse

**Figure 2:**
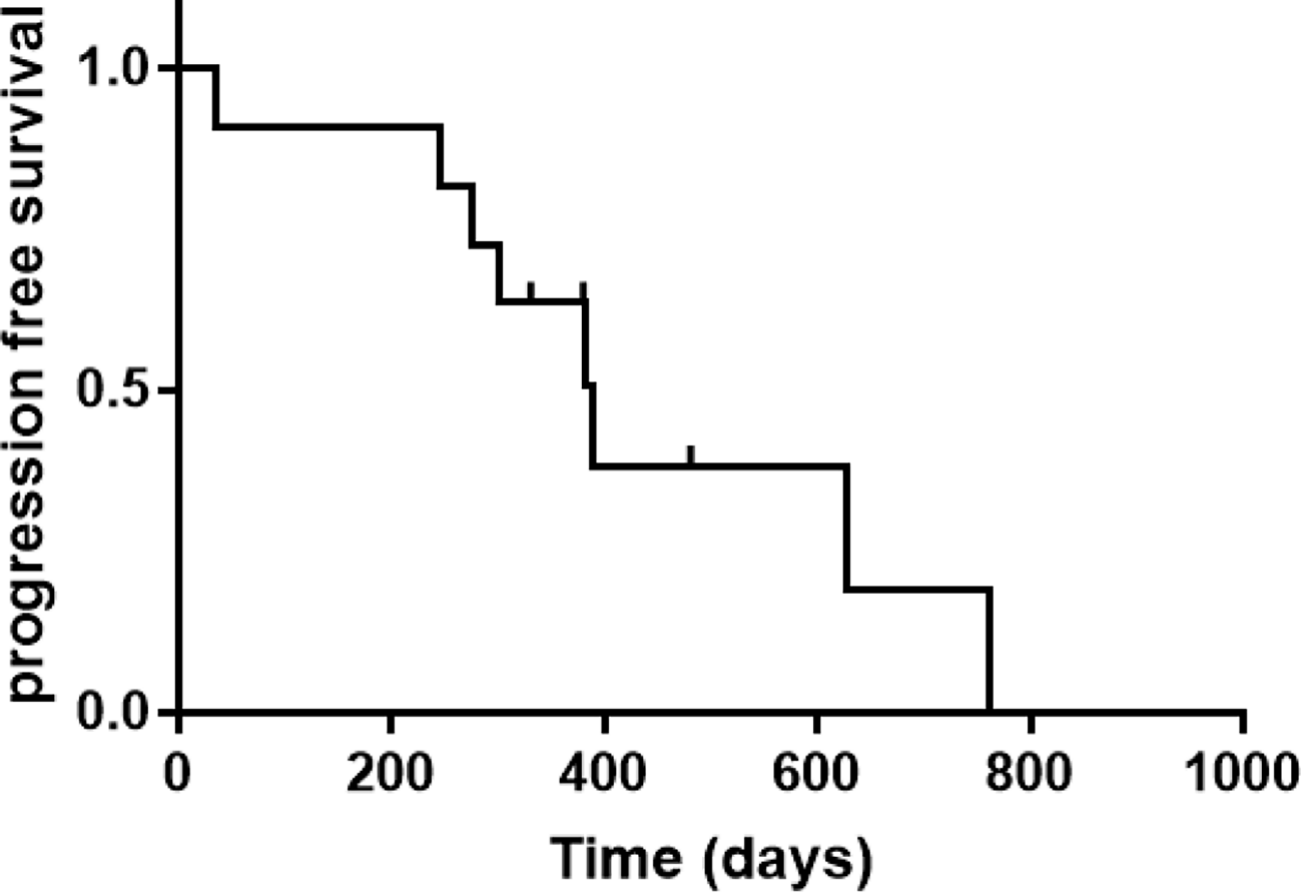
Kaplan-meier curve for survival analysis.

Of 11 patients, 10 achieved CR at the first evaluation and one developed a new lesion in the right precentral gyrus after one treatment cycle. The remaining 10 patients completed the initial induction chemotherapy as per protocol and 9 were referred to the maintenance phase. The median follow-up period was 18.3 (range, 10.5–27.8) months, and 8 of the 11 (72.7%) patients had relapsed at the last follow-up (see **Table 2**). CNS and intraocular relapses occurred in five and three patients, respectively. The median relapse time was 12.7 (range, 1.2–25.4) months (**Figure 2**). All the patients were alive until the last follow-up, and the overall survival (OS) rate was 100%.

### Safety and Toxicity

Safety evaluation was performed using the CTCAE4.0 grading system, and the results are summarized in **Table 3**. Only the worst grade for each patient was reported. Most of the systemic AEs were mild, with only three patients experiencing grade 3 events during the entire follow-up period, and there were no grade 4 AEs. Only two events of grade 3 leukopenia and grade 3 pneumonia occurred in one patient in the maintenance phase.

**Table 3:** Systemic and local adverse events during treatment.

	Grade 1-2 n (%)	Grade 3 n( %)	Grade 4 n (%)
Neutropenia	6(54.5%)	1(9.1%)	0
Anemia	1(9.1%)	0	0
Thrombocytopemia	0	0	0
Infusion reaction	3(27.3%)	1(9.1%)	0
Nausea and vomiting	4(36.4%)	0	0
Hepatobiliary disorders	5(45.5%)	0	0
Renal damage	0	0	0
Rash	3(27.3%)	0	0
respiratory tract infection and pneumonia	3(27.3%)	1(9.1%)	0
Cataract	1(9.1%)	6(54.45)	0
Corneal epitheliopathy	3(27.3%)	0	0

For local complication evaluation, grade 3 cataract events occurred in six patients, and five of six cataracts developed in the eyes for which vitrectomies were performed. The investigators estimated that the cataracts were probably related to the vitrectomy rather than intravitreal MTX injections.

For the neurotoxicity evaluation, headache, dizziness and leg numbness occurred in 2, 1 and 1 patients. All these were Grade 1-2 adverse effects and revealed within 1 months. The investigators estimated these neurotoxicities were probably related with lenalidomide.

### CSF Analysis

All patients had elevated CSF IL-10 concentrations (median, 32.5 pg/mL; IQR, 23.9–102.7) at the time of diagnosis, and eight patients had elevated protein levels (median, 0.55 g/L; IQR, 0.45–0.64). The CSF IL-10 concentration decreased after two or three cycles in all patients, but then they increased in all nine patients who completed six cycles of induction. Six patients eventually relapsed, and the median interval from IL-10 re-increase to clinical relapse was 3 (range, 0–12) months.

## Discussion

PVRL is a rare subtype of PCNSL, and a consistent treatment approach for patients with PVRL is lacking. To the best of our knowledge, this study included one of the largest uniformly treated series of patients with PVRL; moreover, we have introduced for the first time lenalidomide as the first-line treatment for PVRL. An increasing number of studies have used systemic intensive treatments for PVRL, similar to those used for CNSL, based on the hypothesis of CNS relapse prevention benefits. Intensive chemotherapy with or without whole-brain radiation prolonged PFS in patients with CNSL; however, they caused severe systemic AEs (4, 13–15).

Based on the findings of previous studies, we hypothesized that adding an effective and less toxic regimen to local treatment could balance efficacy and safety. Furthermore, published retrospective studies have often included a combination of patients with PVRL and those with VRL and concomitant CNS involvement at diagnosis, making treatment and survival analysis challenging (7, 16, 17). In two relatively large-scale retrospective studies, the PFS ranged from 1.8 to 3.9 years (1, 7). Only five studies included isolated PVRL without CNS lesions and used a uniform treatment regimen (**Table 4**).

**Table 4:** Summary of recent studies of PVRL patients who underwent unified systemic treatment regimen.

Studies	Patient number	Regimen	Median follow-up	PFS	OS	Local toxicity (grade 3-4)	Systemic toxicity (grade3-4)
Taoka et al. (4)	5	R-MPV×5+WBRT(23.4Gy)+ intravitreal MTX	32 months	No patient relapse	No death	NA	100%
Akiyama et al. (18)	10	intravitreal MTX+HD-MTX(3.5g/m^2^)×5	29.5 months	2-year PFS 58.3%	NA	NA	30%
Cheah et al. (13)	11	R-MPV×5+ bilateral globe irradiation (36Gy) +R-AraC×2+IT	4.2 years	Median PFS 3.8 years	NA	6 cataracts (grade 3)	63.6%
Ma et al. (19)	13	HD-MTX(8.0g/m2) twice per week until remission+ intravitreal MTX	40.2 months	Median PFS 19.5 months	Median OS not reached	NA	23.1% (all hepatotoxicity)
de la Fuente et al. (15)	12	bilateral globe irradiation(36Gy)+ MPV ± R×6	68 months	Median PFS not reached	Median OS not reached	Total AE 91%, greater than grade 3 not mentioned	Not mentioned

AE, adverse effects; HD, high-dose; IT, intrathecal injection; MTX, methotrexate; PD, progressive disease; PFS, progression free survival; R, rituximab; R-AraC, rituximab, high-dose cytarbine; R-MPV, rituximab, methotrexate, procarbazine, vincristine; WBRT, whole-brain radiotherapy; WBRT, whole-brain radiotherapy; NA, not Applicable.

Rituximab is widely used to treat B-cell lymphomas and has been used intraocularly for treating PVRL since 2007. Lenalidomide is a multitarget immunomodulator that has a wide range of indications from multiple myeloma to lymphomas. A recent study (REVRI study) showed significant activity against relapsed or refractory (R/R) PCNSL and in PVRL patients with good tolerance. Among the 17 patients who presented with intraocular localization, six (35%) achieved ocular CR (20). We conducted this prospective trial to evaluate the R2 regimen in isolated PVRL.

The safety of the R2 regimen followed by lenalidomide in PVRL was confirmed, and no dose reduction or treatment delay occurred during the treatment. Compared with the MPV (methotrexate, procarbazine, vincristine) regimen, which had severe systemic toxicities (3–4 grade, 63.6–100%) (4, 13), the R2 regimen was associated with only grade 3 AEs (27.3%) and no grade 4 AEs. In addition, ocular complications were reduced significantly following ocular radiation. In the trials involving radiation therapy, local and grade 3 toxicities occurred in 91% and 54.5% patients, respectively. In this study, five of six eyes with grade 3 cataract underwent a vitrectomy, which the investigator estimated was related to the cataracts, rather than the intravitreal injection. Corneal epitheliopathy is a direct complication of intravitreal injection, and only three patients developed grade 1–2 AEs. Furthermore, no treatment-related neurotoxicity was observed in this study.

In this study, 90.1% (10/11) of the patients achieved CR after three cycles of induction therapy (including seven intravitreal injections), except for one patient who developed a new CNS lesion. Frenkel et al. (6) reported that malignant cells were cleared after a median of five (range, 2-11) MTX injections, and this onset time was similar to that observed in our study. However, the duration of response was much shorter than that in previous studies. In 2007, Grimm et al. (1) reviewed 83 patients with intraocular lens and found that 56% patients relapsed with a median PFS of 29 months; moreover, they observed no difference in relapse risk between the two treatment strategies.

Riemens et al. (7) conducted a 17-center European collaborative study in 78 patients with PVRL in 2015, the patients received various combinations of systemic and intrathecal chemotherapy, whole-brain radiotherapy, and peripheral blood stem cell transplantation. CNSL development in 28 of 78 patients was reported at a median follow-up of 49 months. In three recent studies with a uniform regimen, the median PFS was approximately 2 years (13, 15, 18). However, in our study, most relapses (5/8) occurred at approximately 12 (range, 9.2–16.0) months and the median PFS was only 12.7 months. Our hypotheses for this phenomenon was that all 11 patients had elevated CSF IL-10 concentrations. This is because CSF IL-10 concentration is highly sensitive and specific in PCNSL diagnosis, and the concentration decreases after effective treatment.

The elevated CSF IL-10 level suggested that the patients may have already developed subclinical lymphoma in the CNS, which was not substantiated using MRI or CSF examination. Furthermore, MYD88 L265P is an oncogenic mutation not present in non-neoplastic proliferations. In a recent study, the mutation was detected in 17 of 23 patients with VRL aqueous humor and vitreous fluid samples, which is evidence of a malignant neoplasm (21). In our study, we tested MYD88 mutations using next-generation sequencing in five CSF samples, and MYD88 L265P mutations were detected in three (unpublished data). CSF abnormalities strongly suggested invisible lesions in the CNS at the baseline.

The studied by Castellino et al. (16) and Grimm et al. (22) showed that PVRL with concurrent CNS involvement is a strong predictor of shorter PFS, even following treatment with intensive systemic chemotherapy (16, 22). Furthermore, the antitumor effect of lenalidomide in PVRL should be assessed cautiously. Lenalidomide shows good blood-brain barrier penetrability at doses > 15 mg/day (23). However, the concentration of lenalidomide in aqueous humor fluid and vitreous has not been assessed yet. Moreover, we monitored the dynamic change of CSF IL-10 concentrations and found that they slowly increased following initiation of the maintenance treatment. The sustained increase in CSF IL-10 was indicative that lenalidomide monotherapy was not sufficient to control the disease.

Because of the high relapse rate, the study was discontinued in January 2020; thus, the concentrations of lenalidomide in the aqueous humor fluid, vitreous, or both still need to be analyzed. Our study had some limitations such as the case number being less than the expectation and a median follow-up period of only 18.3 months, which was too short to evaluate the proposed endpoint. These limitations were mainly caused by the unexpected relapse rate.

In conclusion, our study demonstrated that adding an R2 regimen to intravitreal MTX injection is a safe option in newly diagnosed patients with PVRL. However, this trial could not reveal the superiority of this strategy over intensive systemic chemotherapy. CSF IL-10 monitoring may be useful in detecting relapses earlier. More bench work is required to confirm the antitumor effect of lenalidomide in PVRL.

## Data Availability Statement

The original contributions presented in the study are included in the article/supplementary material. Further inquiries can be directed to the corresponding authors.

## Ethics Statement

The studies involving human participants were reviewed and approved by Ethical Committee of Peking Union Medical College Hospital. The patients/participants provided their written informed consent to participate in this study.

## Author Contributions

Contribution: DBZ, WZ, and MZ designed the study. YZ, XZ, DMZ, JY, WW, and DQZ acquired, analyzed, or interpreted the data. LZ, XW, and CJ conducted laboratory and pathological tests. YZ performed statistical analyses and wrote the manuscript. WZ and MZ critically revised the manuscript for important intellectual content. DBZ, WZ, and MZ supervised the study. All authors contributed to the article and approved the submitted version.

## Funding

This study was funded by the CAMS Innovation Fund of Medical Sciences (CIFMS) 2019-I2M-2-009 and CIFMS 2016-12M-1-001.

## Conflict of Interest

The authors declare that the research was conducted in the absence of any commercial or financial relationships that could be construed as a potential conflict of interest.
